# Genomic Surveillance of *Streptococcus pyogenes* Strains Causing Invasive Disease, United States, 2016–2017

**DOI:** 10.3389/fmicb.2020.01547

**Published:** 2020-07-24

**Authors:** Yuan Li, Joy Rivers, Saundra Mathis, Zhongya Li, Srinivasan Velusamy, Srinivas A. Nanduri, Chris A. Van Beneden, Paula Snippes-Vagnone, Ruth Lynfield, Lesley McGee, Sopio Chochua, Benjamin J. Metcalf, Bernard Beall

**Affiliations:** ^1^Respiratory Diseases Branch, Division of Bacterial Diseases, National Center for Immunization and Respiratory Diseases, Centers for Disease Control and Prevention, United States Department of Health and Human Services, Atlanta, GA, United States; ^2^Minnesota Department of Health, St Paul, MN, United States

**Keywords:** group A streptococcus, genomics, surveillance studies, vaccines, antibiotic resistance, virulence

## Abstract

**Background:**

*Streptococcus pyogenes* is a major cause of severe, invasive infections in humans. The bacterial pathogen harbors a wide array of virulence factors and exhibits high genomic diversity. Rapid changes of circulating strains in a community are common. Understanding the current prevalence and dynamics of *S. pyogenes* lineages could inform vaccine development and disease control strategies.

**Methods:**

We used whole-genome sequencing (WGS) to characterize all invasive *S. pyogenes* isolates obtained through the United States Center for Disease Control and Prevention’s Active Bacterial Core surveillance (ABCs) in 2016 and 2017. We determined the distribution of strain features, including *emm* type, antibiotic resistance determinants, and selected virulence factors. Changes in strain feature distribution between years 2016 and 2017 were evaluated. Phylogenetic analysis was used to identify expanding lineages within *emm* type.

**Results:**

Seventy-one *emm* types were identified from 3873 isolates characterized. The *emm* types targeted by a 30-valent M protein-based vaccine accounted for 3230 (89%) isolates. The relative frequencies of *emm* types collected during the 2 years were similar. While all isolates were penicillin-susceptible, erythromycin-resistant isolates increased from 273 (16% of 2016 isolates) to 432 (23% of 2017 isolates), mainly driven by increase of the *erm*-positive *emm* types 92 and 83. The prevalence of 24 virulence factors, including 11 streptococcal pyrogenic toxins, ranged from 6 to 90%. In each of three *emm* types (*emm* 49, 82, and 92), a subgroup of isolates significantly expanded between 2016 and 2017 compared to isolates outside of the subgroup (*P*-values < 0.0001). Specific genomic sequence changes were associated with these expanded lineages.

**Conclusions:**

While the overall population structure of invasive *S. pyogenes* isolates in the United States remained stable, some lineages, including several that were antibiotic-resistant, increased between 2016 and 2017. Continued genomic surveillance can help monitor and characterize bacterial features associated with emerging strains from invasive infections.

## Introduction

Group A *Streptococcus* (GAS) is a Gram-positive bacterium that causes a variety of diseases in humans. Invasive GAS (iGAS) infections are associated with high case fatality ratios. Clinical manifestations of iGAS disease include cellulitis, bacteremia, pneumonia, necrotizing fasciitis, and streptococcal toxic shock syndrome. Outbreaks of iGAS disease can occur and have been reported in residents of long-term care facility (Deutscher et al., 2011; Dooling et al., 2013; Ahmed et al., 2018; Nanduri et al., 2019), in people experiencing homelessness (Bundle et al., 2017; Adebanjo et al., 2018; Mosites et al., 2018), and in people who inject drugs (Lamagni et al., 2008; Kwiatkowska et al., 2018). Frequency of predominant GAS strains in a community often vary over time, presumably due to changes in population-level immunity against certain strains (Vlaminckx et al., 2005; Stockmann et al., 2012). The introduction of a novel or rare strain into a population has repeatedly been demonstrated to cause a rise in *Streptococcus pyogenes* infections (Tyrrell et al., 2010; Turner et al., 2015; Mosites et al., 2018), and key genomic changes within relatively uncommon strains that affect virulence factor structure or expression have been associated with subsequent global expansion (Turner et al., 2015; Beres et al., 2016). Although stable between 2000 and 2012, the incidence of invasive GAS infections in the United States (Nelson et al., 2016; CDC, 2019) nearly doubled from a baseline of 3.4–4.3 per 100,000 population to 7.26 cases per 100,000 persons in 2017 (CDC, 2019). Understanding iGAS disease trends and associated strain features through surveillance increases the understanding of population dynamics of GAS and can guide development of effective prevention strategies.

The GAS genome encodes a wide array of virulence factors. The M protein, encoded by the *emm* gene, is a major virulence factor involved in resisting phagocytosis (Fischetti, 1989) and elicits type-specific antibodies; this antigen is the foundation of the 30-valent M protein-based vaccine currently under development (Dale et al., 2011). The high *emm* gene sequence diversity within the *S. pyogenes* species formed the basis for the most-widely used strain subtyping method, *emm*-typing, with over 240 *emm* types documented to date (Bessen et al., 2018). The *erm* family antibiotic resistance genes in *S. pyogenes* are carried by several different transposon elements and shows evidence of intra-species spreading (Brenciani et al., 2007). Numerous exotoxin genes in *S. pyogenes* encode superantigens that stimulate uncontrolled T-cell responses underlying streptococcal toxic shock syndrome (Fraser and Proft, 2008). In the United States, approximately 40–60% of *S. pyogenes* isolates contain an active *sof* gene (Chochua et al., 2017). The presence of *sof* can be predicted from *emm* gene sequence type (Facklam et al., 2002; Smeesters et al., 2010) and genetic organization of *emm* and neighboring “*emm*-like” genes (Bessen and Hollingshead, 1994). These two broad categories of GAS, distinguished by *sof*, *emm* pattern (Sanderson-Smith et al., 2014), or *emm*-locus gene organization (Bessen et al., 1996) differ in disease seasonality (Chochua et al., 2017; Smeesters et al., 2017), distributions between tropical/non-tropical locations, and exhibit different tissue preferences (Bessen et al., 2018). Recent developments in whole-genome sequencing (WGS) technology allow a fairly detailed characterization of *S. pyogenes* strain features, including *emm* type, antibiotic resistance genes, exotoxin genes, and other virulence factors for a population (Chochua et al., 2017). Use of WGS to characterize iGAS isolates collected from longitudinal surveillance will provide invaluable information on population dynamics, vaccine target prevalence, virulence factor distributions, and strain features associated with emerging lineages. In this study we aimed to determine the distribution of critical *S. pyogenes* strain features in a well-defined United States population and monitor changes from 2016 to 2017.

## Materials and Methods

### Surveillance and Isolate Collection

All iGAS isolates were identified through the Active Bacterial Core surveillance (ABCs) as previously described (Chochua et al., 2017). ABCs is active, laboratory- and population-based surveillance of invasive bacterial infections as part of the Emerging Infections Program Network (EIP) of the United States Centers for Disease Control and Prevention (CDC). The iGAS surveillance areas represented approximately 34.2 million residents in 10 sites in 2017 (CDC, 2019). ABCs staff routinely contact microbiology laboratories serving hospitals at each site to identify cases of iGAS disease. A case is defined as illness with isolation of GAS from a normally sterile site or from a wound culture accompanied by necrotizing fasciitis or streptococcal toxic shock syndrome in a resident of a surveillance site. All available GAS isolates are sent to CDC’s *Streptococcus* laboratory for characterization.

In this study we characterized case isolates identified between January 01, 2016 and December 31, 2017. We limited our analyses to *S. pyogenes* (99.4% of GAS isolates); non-*S. pyogenes* GAS isolates (e.g., group A *Streptococcus dysgalactiae* subspecies *equisimilis*) have been previously described and were excluded (Chochua et al., 2019).

### Whole-Genome Sequencing Analysis

Whole-genome sequencing was performed according to the method previously reported (Chochua et al., 2017). Briefly, each isolate was colony-purified, and the genomic DNA sample was extracted from an overnight broth culture. A multiplexed sequencing library was prepared from equal mixing of 48 genomic DNA samples. The library was sequenced on the Illumina MiSeq platform to produce 250-bp, paired-end reads with expected coverage depth of >50× for each isolate. Resulting sequencing reads were processed by a validated bioinformatics pipeline (Chochua et al., 2017) to obtain draft whole genome assembly (contigs), to identify subtype [*emm* type and multi-locus sequence type (ST)], to infer antimicrobial susceptibility, and to determine strain features for each isolate. The bioinformatics pipeline analysis output of each isolate was manually curated, and the curated results were entered into a MySQL database management system. The MySQL database was queried on January 10, 2019 to retrieve WGS analysis results of the study isolates. Bioinformatics scripts and associated sequence databases are available at https://github.com/BenJamesMetcalf.

### Estimation of Vaccine Coverage

A 30-valent M protein-based vaccine candidate (Dale et al., 2011) composed of antigenic peptides from M proteins of 30 different *emm* types is a leading vaccine candidate currently in human trials (Vekemans et al., 2019). An *S. pyogenes* isolate was considered covered by the M protein-based vaccine candidate if the isolate belonged to one of the 30 *emm* types. Another antigen, the M-related protein (Mrp), has been proposed as a next generation GAS vaccine (Courtney et al., 2017); the addition of the additional Mrp antigen to M protein–based vaccines would broaden global coverage. Mrp antibodies are shown to be acquired following natural infection in the same age-related manner as M protein antibodies. An *S. pyogenes* isolate was considered as covered by the M-related proteins-based vaccine candidate if the isolate was positive for the *mrp* gene target (Chochua et al., 2017).

### Antimicrobial Resistance Determinants

The WGS-analysis pipeline screened for the presence of streptococcal antimicrobial resistance determinants (gene alleles), which included 21 targets in an in-house sequence database (Chochua et al., 2017), 1913 targets in the Resfinder sequence database (Zankari et al., 2012), and 1653 targets in the ARG-ANNOT sequence database (Gupta et al., 2014). The presence of the antimicrobial resistance genes or mutations was used in a “rules-based” resistance classification of a strain to perform a WGS-based antimicrobial susceptibility test (AST) as previously described (Chochua et al., 2017). WGS-based AST quality control was performed by comparing to conventional phenotypic, culture-based AST (see Supplementary Method for detail). A full list of antibiotic resistance targets observed in the study isolates is shown in Supplementary Table S1. The four common antimicrobial resistance targets analyzed in this study were:

ERM: presence of *erm* family genes associated with macrolide and clindamycin resistance.
TET: presence of *tet* family genes associated with tetracycline resistance.
PARC_GYRA : presence of *parC*/*gyrA* mutations associated with fluoroquinolone resistance.
MEF: presence of *mef* family genes associated with macrolide resistance.

Additionally, a PBP2x typing scheme was incorporated that detects substitutions within the transpeptidase region of PBP2x^21^.

### Phylogenetic Analysis

Phylogenetic analysis was performed separately for each individual *emm* type that contained 20 or more isolates. Draft assemblies of isolates were first aligned by a fast core-genome multi-aligner implemented in Parsnp v1.2 (Treangen et al., 2014) with the -x option to filtering out regions of recombination. The core genome alignment generated by Parsnp was used to build a phylogenetic tree using FastTree v2.1.10 (Price et al., 2009). All monophyletic groups in the phylogenetic tree were identified using the subtree function implemented in the R ape package (R.C.Team, 2015). Isolates belonging to a monophyletic group were defined as a subgroup. To identify an expanded subgroup, the relative frequency of the subgroup in 2016 was compared to that in 2017 using a Fisher’s exact test and a *p*-value less than the Bonferroni-corrected threshold for multiple testing was considered significant. If two subgroups were both significantly expanded and one subgroup contained all isolates of the other subgroup, then only the subgroup with smaller *p*-value was retained for further analysis.

To identify genome sequence features that were characteristic for a subgroup, draft assemblies were converted into *k*-mers using the fsm-lite software, which enabled frequency-based string mining (Lees et al., 2016). A characteristic *k*-mer of subgroup X was defined as a *k*-mer that was present in >90% of isolates within subgroup X but in <10% of isolates outside subgroup X, or present in <10% of isolates within subgroup X but in >90% of isolates outside subgroup X. Annotation of characteristic *k*-mers were performed by mapping the *k*-mer sequences against reference, complete GAS genomes using miniMap (Li, 2016).

### Statistical Analysis

Test of equal proportions in two groups was performed using the Fisher’s exact test. *P*-values were adjusted for multiple comparisons using the Bonferroni method where indicated. The strength of the association between *emm* type and strain features was quantified by the Cramer’s *V* statistic. All statistical analysis was performed in the R software v3.2.2 (R.C.Team, 2015).

## Results

### Distribution of *emm* Types and STs

**FIGURE 1:**
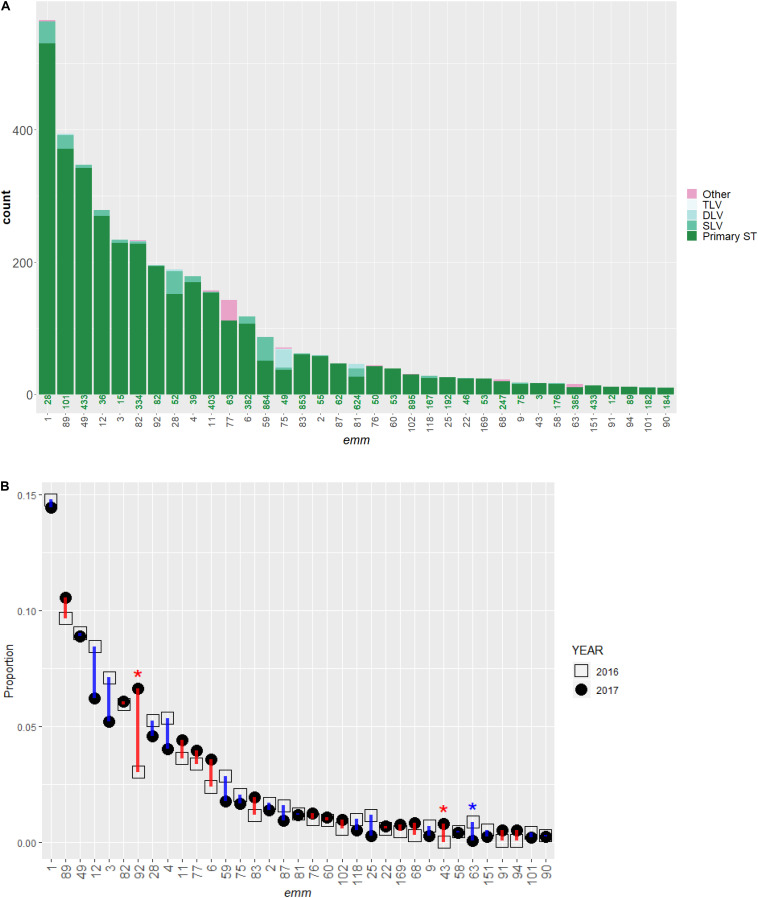
Distribution of *emm* types and multi-locus sequence type (ST) in invasive *S. pyogenes* isolates identified through the Active Bacterial Core surveillance in 2016 and 2017. **(A)** A bar plot of isolate count. Each bar represents an *emm* type and is colored according to ST. The green number under each bar indicates the most frequently observed ST (primary ST) within the *emm* type. The single, double, or triple-locus variants of the primary ST are indicated by SLV, DLV, and TLV, respectively. The magenta color indicates isolates differing from the primary ST by at least four of the seven loci used to define a ST. **(B)** Change in relative frequency of *emm* types between 2016 (open square) and 2017 (solid circle). The proportion of isolates belong to the *emm* type in all isolates of that year (*n* = 1,716 for year 2016 and 2,157 for year 2017) is shown. The vertical bars indicate an increase (red) or decrease (blue) in proportion. An asterisk (*) indicates the relative frequency of an *emm* type differed significantly between the 2 years (Fisher’s exact test, *p* < 0.00070). Only *emm* types containing 10 or more isolates are shown.

The ABCs identified 4,425 iGAS cases between January 01, 2016 and December 31, 2017. GAS isolates from 3,895 (88%) cases were available and characterized by WGS. We included 3,873 (99.4%) isolates identified as *S. pyogenes*; 22 non-*S. pyogenes* GAS isolates (all group A *Streptococcus dysgalactiae* subspecies *equisimilis*) were excluded. Table 1 shows the distribution of strain features inferred from WGS. A total of 71 *emm* types were identified among the 3,864 isolates (9 isolates were non-typeable). The number of isolates within each *emm* type ranged from 1 (for 20 *emm* types) to 566 (*emm1*). The 10 most common *emm* types (in decreasing order: 1, 89, 49, 12, 3, 82, 92, 28, 4, and 11) each contained ≥58 isolates and collectively accounted for 2,773 isolates (72%). More than 97% of isolates (*n* = 3,773) corresponded to the 31 most common *emm* types that each represented at least 10 isolates (Figure 1A).

**TABLE 1:** Prevalence of surface structure and virulence related strain features in invasive *S. pyogenes* isolates identified through the Active Bacterial Core surveillance system in the United States in 2016 and 2017 (*n* = 3873).

Strain feature^a^	Description	Count (proportion)
SPE_G	Streptococcal pyrogenic exotoxin G	3,500(90.4%)
FBAA	Fibronectin-binding protein of group A streptococci type A	3,204(82.7%)
SME_Z	Streptococcal mitogenic exotoxin	3,123(80.6%)
D330G	NADase D330G substitution associated with increased NADase activity	3,038(78.4%)
CAPSULE	*hasA* hyaluronic acid synthetase operon determinant	2,650(68.4%)
MRP	*emm*-like *mrp* virulence gene	2,609(67.4%)
ENN	*emm*-like *enn* virulence gene	2,565(66.2%)
SOF	Serum opacity factor	2,515(64.9%)
PRTF2	*S. pyogenes* fibronectin-binding protein	2,477(64%)
SFB1	*S. pyogenes* fibronectin-binding protein I	2,424(62.6%)
SPE_C	Streptococcal pyrogenic exotoxin C	2,019(52.1%)
PNGA3	Pnga 3 – Clade 3 up-regulated promoter of the *nga* operon	1,865(48.2%)
SPE_J	Streptococcal pyrogenic exotoxin J	1,190(30.7%)
SPE_H	Streptococcal pyrogenic exotoxin H	1,003(25.9%)
SPE_I	Streptococcal pyrogenic exotoxin I	954(24.6%)
SDA1	Virulence associated DNAse	890(23%)
SIC	Streptococcal inhibitor of complement	826(21.3%)
SPE_A	Streptococcal pyrogenic exotoxin A	788(20.3%)
SS_A	Streptococcal Superantigen A	510(13.2%)
SPE_K	Streptococcal pyrogenic exotoxin K	496(12.8%)
R28	Homolog of a group B streptococcal adhesin	365(9.4%)
SPE_L	Streptococcal pyrogenic exotoxin L	269(6.9%)
SPE_M	Streptococcal pyrogenic exotoxin M	263(6.8%)
ROCA	*rocA* null mutation conserved within type *emm3* strains	237(6.1%)

*^*a*^A strain feature is either positive (presence) or negative (absence) in an isolate.*

A total of 143 STs were assigned for 3,845 isolates while STs for the other 28 isolates (0.72%) were unassigned at the time of database query. Isolates within a given *emm* type were almost exclusively represented by a primary ST (the most frequently observed ST within an *emm* type) and its single, double, or triple-locus variants (Figure 1A). Isolates with an ST that differed from the within-*emm* type primary ST by four or more loci (Figure 1A, red bars) were only seen in 64 isolates (1.7%), approximately half of which (*n* = 31) were type *emm77*. Each of the 10 most common STs (in decreasing order: 28, 101, 433, 36, 334, 15, 82, 39, 403, and 52) were also the primary ST of one of the respective 10 most common *emm* types (1, 89, 49, 12, 3, 82, 92, 28, 4, and 11). The 10 and 20 most common STs represented 2664 (69%) and 3081 (85%) isolates, respectively. The *S. pyogenes* ST was strongly correlated with the *emm* type (Cramer’s *V* = 0.93). Within each ST, the number of *emm* types observed was either one (in 135 STs), or two (in 8 STs).

In 2016 and 2017, 1,716, and 2,157 isolates were identified, respectively. The relative frequency of common *emm* types in 2016 was similar to that in 2017 (Figure 1B) with three significant exceptions: *emm*92, 63, and 43 (Fisher’s exact test, *P*-values < 0.001). The proportion of *emm*92 isolates increased from 3.0% (*n* = 52) in 2016 to 6.7% (*n* = 143) in 2017 and occurred primarily in three states: Colorado, Tennessee, and New Mexico (Supplementary Figure S1A). The proportion of *emm*63 isolates decreased from 0.9% (*n* = 15) to <0.1% (*n* = 1); the decrease was ubiquitous across all five states where *emm*63 was present in 2016 (Supplementary Figure S1B). In four states the proportion of *emm*43 isolates increased from 0% (*n* = 0) to 0.8% (*n* = 17) (Supplementary Figure S1C).

### Estimated Vaccine Coverage

Twenty-four of the 30 *emm* types covered by the M protein-based vaccine candidate were found in 3,411 (88%) of the 3,873 study isolates. WGS identified the presence of the Mrp target in 2,609 (67%) isolates (Table 1), which included nearly all (409 of 462) isolates not targeted by the 30-valent M protein-based vaccine candidate. The combination of antigens found in both vaccine candidates could elicit protection from infection due to 98.6% of invasive *S. pyogenes* isolates in this study.

### Major Genetic Determinants to Antimicrobial Resistance

**FIGURE 2:**
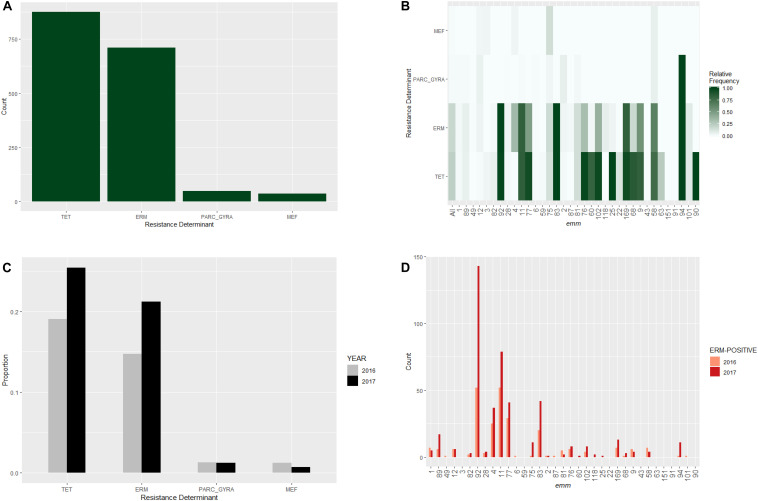
Distribution of major antibiotic resistance determinants in invasive *S. pyogenes* isolates identified through the Active Bacterial Core surveillance in 2016 and 2017. **(A)** A bar plot of isolates positive for the *erm* genes (ERM), the *mef* genes (MEF), the *tet* genes (TET), or mutations in the *parC* and *gyrA* genes associated with fluoroquinolone non-susceptibility (PARC_GYRA). **(B)** Relative frequency of antibiotic resistance determinant. The color scale indicates the proportion of isolates that were positive indicated resistance determinant within an *emm* type. “All” represents all *emm* types combined. **(C)** Prevalence of resistance determinants for isolates identified in 2016 (gray bars) and 2017 (black bars). **(D)** Number of ERM-positive isolates identified in 2016 (pink bars) and 2017 (red bars) within individual *emm* type. Only *emm* types containing 10 or more isolates are shown.

Among the 3,873 isolates, the most frequently identified resistance determinants were the TET target in 876 (22.6%) isolates, the ERM target in 711 (18.4%) isolates, the MEF target in 36 (0.9%) isolates, and the GYRA_PARC target in 49 (1.4%) isolates (Figure 2A and Supplementary Table S1). The ERM target identified in the study isolates included *ermB* (*n* = 219), *ermT* (*n* = 276), *ermTR* (*n* = 211), and unassigned *erm* genes (*n* = 5, Supplementary Table S1). Seven ERM-positive isolates carried additional changes associated with erythromycin/clindamycin resistance (Supplementary Table S1), including 23S rRNA mutations (*n* = 3), *lsa* gene (*n* = 2), and *lsac* gene (*n* = 2). Most of the *gyrA*/*parC* gene mutations encoded the PARC-S79F (*n* = 42) substitution associated with intermediate levofloxacin resistance (MIC = 4 μg/mL). Only four isolates carried mutations in both *gyrA* and *parC* and were levofloxacin-resistant (MIC ≥ 8 μg/mL, Supplementary Table S1).

No PBP2x sequence types or other determinants associated with penicillin non-susceptibility were identified (Supplementary Table S1). Approximately (79% 3,220/4,095) of isolates were of the reference sequence type pBP2x-1, similar to the percentage (76.1%) found during 2015. The second most common type (PBP2x-3) was observed among 396 isolates, all of which were type *emm89*. Although 31 additional PBP2x types were each observed among 1–64 isolates, none were associated with non-susceptibility to beta lactam antibiotics, however, it is possible that our conventional testing panel for six different beta lactam antibiotics did not contain antimicrobial concentrations low enough to detect low level changes above basal wild type MICs. One previously described PBP2x substitution reported to be associated with very small increases of MICs for penicillin and ampicillin, P601L (PMID: 31996443), was observed among 10 isolates. Four isolates (3 *emm75* and 1 *emm1*) contained PBP2x-7 (P601L). Six *emm89* isolates contained PBP2x-16 (S562T + P601L), from 2015 isolates in our previous study we note that we have encountered a third type, PBP2x-28 (M593V, P601L) from a single type *emm87* isolate (Fraser and Proft, 2008). No gene alleles associated with reduced susceptibility to vancomycin, chloramphenicol, linezolid, trimethoprim-sulfamethoxazole, or synercid were found (Supplementary Table S1). Only one isolate was non-susceptible to rifampin (MIC = 6 μg/mL) associated with an *rpoB* mutation resulting in the RPOB-D476E change (Supplementary Table S1).

Major antimicrobial resistance determinants showed strong association with relatively infrequent *emm* types (Figure 2B). Greater than 80% of isolates belonging to *emm* types 92, 11, 83, 169, and 94 had an erythromycin resistance determinant (Figure 2B). All 12 isolates belonging to *emm94* carried the PARC-S79F substitution which is a levofloxacin resistance determinant (Figure 2B). Major resistance determinants were rarely seen in the six most common *emm* types (1, 89, 49, 12, 3, and 82; Figure 2B).

We next investigated whether the relative frequency of common resistance determinants differed between years (Figure 2C). The proportion of ERM-positive isolates in 2017 (21%) was significantly higher than that in 2016 (15%; Fisher’s exact test, *P*-value = 0.001). A similar increase was observed for the proportion of TET-positive isolates (25% vs. 19%; *P*- = 0.001). In contrast, no significant between-year difference in the relative frequency of MEF-positive or GyrA/ParC substitution mutants was found (*P*-values > 0.05). The prevalence of erythromycin-resistance (isolates positive for ERM or MEF target) increased from 16% of 2016 isolates to 22% of 2017 isolates. The largest increase in ERM-target positive isolates was due to the increases of the predominantly resistant *emm* types 92 (*ermT*), 83 (*ermTR*), and 11 (*ermTR*) (Figure 2D).

### Surface Structure and Virulence Related Strain Features

**FIGURE 3:**
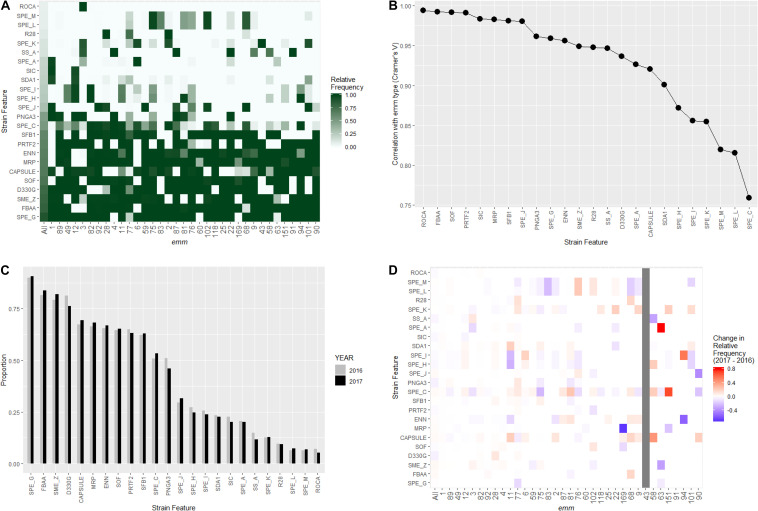
Distribution of virulence related strain features in invasive *S. pyogenes* isolates identified through the Active Bacterial Core surveillance in 2016 and 2017. **(A)** Relative frequency of strain feature by *emm* type. The color scale indicates the proportion of isolates that were positive for indicated strain feature within an *emm* type. “All” represents all *emm* types combined. **(B)** Correlation between *emm* type and each strain feature. The *x*-axis is ordered by decreasing association strength (Cramer’s *V*). **(C)** Prevalence of strain features for isolates identified in 2016 (gray bars) and 2017 (black bars). **(D)** Change of strain feature relative frequency within individual *emm* type. The color scale of each tile represents the difference in within-*emm* relative frequency of a strain feature between 2016 and 2017. Red indicates an increase while blue indicates a decrease. The within-*emm* relative frequency could not be calculated for *emm43* in 2016 because no emm43 isolate was observed in that year.

The WGS-analysis pipeline screened for the presence of 13 surface structure and virulence factor gene targets and 11 streptococcal pyrogenic exotoxin gene targets in each isolate (Table 1). The prevalence of different strain features among the 3,873 isolates ranged from 237 (6.1%) for the *rocA* null mutation seen only in *emm3* strains to 3,500 (90%) for streptococcal pyrogenic exotoxin G (Table 1). Strain features showed strong association with *emm* type (Cramer’s *V* range 0.76–0.95; Figure 3B). Approximately one-third of the study isolates (*n* = 1223) were predicted to lack the hyaluronic acid capsule by virtue of lacking the *hasA* target [e.g., being nearly all *emm4* and *emm22* isolates (Flores et al., 2012)], or by containing inactivating frameshifts within *hasA* (*emm28* is an example where all isolates contained a conserved frameshift within *hasA*) (Turner et al., 2019). It is interesting to note the fairly common occurrence of what appear to be sporadically occurring null mutations within *hasA* that occur within many predominantly encapsulated GAS (data not shown).

None of the 24 strain features showed significant between-year difference in the relative frequency (Figure 3C, Fisher’s exact test, *p*-values > 0.05 after Bonferroni correction). A close examination of the yearly change in strain feature relative frequency within individual *emm*-type identified several notable variations (Figure 3D). For example, within *emm*11, the proportion of isolates positive for the virulence associated DNAse (SDA1) increased from 3.2% (2 of all 62 *emm*11 isolates in 2016) to 20.2% (17 of all 84 *emm*11 isolates in 2017). Within *emm*6, the proportion of isolates positive for the streptococcal pyrogenic exotoxin I (SPE_I) increased from 12% (5/41) to 30% (22/74). Within *emm*3, the proportion of isolates positive for the streptococcal pyrogenic exotoxin C (SPE_C) decreased from 17% (21/122) to 7% (8/110).

### Expanded Lineages Within *emm* Type

**FIGURE 4:**
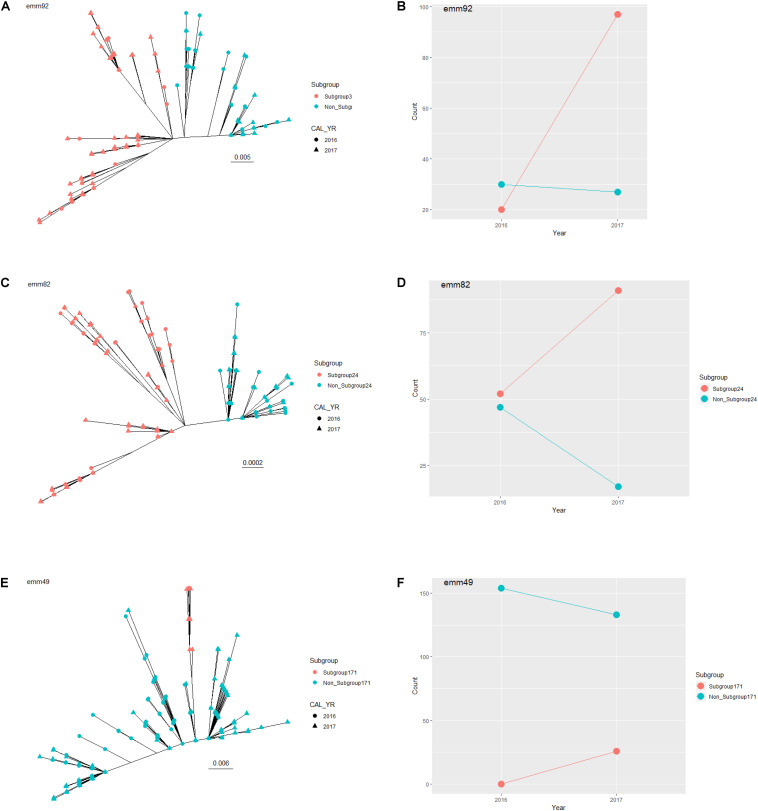
Expanding subgroups within *emm* type between 2016 and 2017. Unrooted maximum-likelihood trees are shown for all *emm92* isolates **(A)**, all *emm82* isolates **(C)**, and all *emm49* isolates **(E)**, identified through the Active Bacterial Core surveillance in 2016 and 2017. The expanding subgroup isolates are colored in red. The scale bar indicates expected change per site. Counts of expanding subgroup isolates (red) and non-expanding subgroup isolates (blue) in 2016 and 2017 are shown for *emm92* **(B)**, *emm82* **(D)**, and *emm92* **(F)**.

We used a phylogenetic approach to investigate whether the relative frequency of a subgroup (lineage) within an *emm* type increased from 2016 to 2017. A subgroup was defined as a single branch that consisted of a common ancestor (the ancestral node) and all its lineal descendants on an *emm*-specific core genome tree. A total of 3,236 subgroups were identified from the 26 *emm* types which contained 20 or more isolates. Three subgroups showed a significant expansion from 2016 to 2017 (Figure 4), including subgroup32 in *emm92* (Figures 4A,B), subgroup25 in *emm82* (Figures 4C,D), and subgroup171 in *emm49* (Figures 4E,F).

Based on all characteristic *k*-mers identified, the subgroup32 of *emm*92 differed from non-subgroup32 emm92 by a sequence variation at a position corresponding to position 995380 of the reference genome NC_003737.2 (M1 SF370 complete genome). Nucleotide sequence at this position was C for all the subgroup32 isolates and A for all the non-subgroup32 isolates. The C to A sequence variation represented a modifier variant upstream of a putative pyridoxamine kinase gene (RefSeq:NP_269347.1). Within *emm92*, the proportion of subgroup32 isolates increased from 0.39 (20 of 52 isolates) in 2016 to 0.76 (109 of 143 isolates) in 2017(Figure 4B; *p*-value = 2.1 × 10^–6^, Fisher’s Exact test; Bonferroni-corrected threshold: 0.05/3236 = 1.5 × 10^–5^).

The subgroup25 of *emm*82 differed from non-subgroup25 of *emm*82 by a sequence variation in the fibronectin binding protein gene (*prtF2*, YP_279582.1), among others (Supplementary Table S2). Nucleotide sequence at *prtF2* position 751 was A for all the subgroup25 isolates and G for all the non-subgroup25 isolates. The A to G sequence variation resulted in a Thr to Ala alteration at amino acid position 251 of the prtF2 protein. Within emm82, the proportion of subgroup25 isolates increased from 0.53 (52/102) in 2016 to 0.81 (106/131) in 2017 (Figure 4D; *p*-value = 1.4 × 10^–6^).

The subgroup171 of *emm*49 differed from non-subgroup171 of *emm*49 by a sequence variation in the branched-chain amino acid aminotransferase gene (*bcaT*, YP_598381.1), among others (Supplementary Table S3). All the subgroup171 isolates had a nucleotide sequence of A at *bcaT* gene position 97 while all the non-subgroup171 isolates had a sequence of G. This A to G sequence variation resulted in a Ser to Gly alteration at amino acid position 33 of the BcaT protein. Within emm 49, the proportion of subgroup171 isolates increased from 0 (0/155) in 2016 to 0.16 (30/192) in 2017 (Figure 4E; *p*-value = 1.4 × 10^–6^).

## Discussion

In this study we characterized the genomic features of 3873 *S. pyogenes* isolates recovered from a population-based surveillance system for invasive GAS infections operating in 10 geographically disparate United States sites in 2016–2017. For the vast majority of the GAS isolates, *emm* type appeared to be a very good approximation of clonal complex, defined by a founder ST and all STs sharing four or more identical ST loci with the founder ST (Feil et al., 2004). Presence of an *emm* type in two or more clonal complexes remained rare except for *emm*77, unlike the much more complex serotype-clonal relationship observed in another human-specific pathogen, *Streptococcus pneumoniae* (Beall et al., 2018). The overall frequency distribution of *emm* types in both years was similar (Figure 1B), which reflected a lack of substantial population structure shift among common *emm* types during this 2-year time period. Significant changes in relative frequency was observed only for *emm* types that are normally rare within ABCs (*emm* 92, 43, and 63), which could result from either directional selection forces acting upon these types or a temporary neutral drift that tended to influence types with smaller effective population size more profoundly. The GAS vaccine candidates based on M- and M related- protein showed high theoretical coverage, consistent with results from previous studies (Dale et al., 2011; Chochua et al., 2017; Courtney et al., 2017). The hypervariable region of the M protein was shown to contain epitopes that both elicit protective antibodies against GAS infection and minimally evoke human tissue cross-reactive antibodies (Dale et al., 1993, 2011; Dale and Walker, 2020). In a recent phase I clinical study, the 30-valent M protein-based vaccine was shown to be safe and immunogenic without eliciting autoimmunity in adults (Pastural et al., 2020).

The WGS-based monitoring system based on PBP2x transpeptidase sequence types automatically flags all GAS strains with *pbp2x* mutations in the routine surveillance (Chochua et al., 2017) as well as in outbreak investigations (Mosites et al., 2018; Vannice et al., 2019). Recently, two nearly isogenic strains were flagged by the CDC’s bioinformatics pipeline and showed substantially elevated ampicillin and amoxicillin MICs (0.125–0.25 μg/ml), that are the highest GAS MICs recorded to date for these antibiotics (Vannice et al., 2019). Both isolates carried a same point mutation within the *pbp2x* leading to a PBP2x T553K substitution (Vannice et al., 2019) and would not have been phenotypically tested if not for the PBP2x point mutation that was detected through our established pipeline (Chochua et al., 2017). The WGS-based monitoring system supports a more sensitive level of surveillance on reduced beta lactam susceptibility and allows further investigations on potential selective advantages conferred by such mutations. PBP2x substitutions found in the recent studies (Musser et al., 2020; Olsen et al., 2020) of *emm* 1, 28, and 89 strains that showed modestly elevated beta lactam MICs, are also represented within this sampling. As shown this year 2016–2017 strain survey and the previous survey of 2015 ABCs GAS (Chochua et al., 2017), the P601L substitution was found among additional *emm* types and within three different PBP types, however, our MIC determinations were not low enough to discern these relatively subtle MIC increases. We now plan to examine our PBP2x type variants carefully to determine exact phenotypic changes that have occurred and to continue to carefully monitor these variants. Although it does appear that *pbp2x* point mutants regularly appear in our surveillance as a consequence of beta lactam selective pressure, present data suggests that there are strict constraints upon sequence variation (Hayes et al., 2020).

Two of the major antibiotic resistance determinants, the ERM and TET targets, were present in approximately 20% of isolates, yet were not evenly distributed among *emm* types (Figure 2B). Instead, the presence of ERM and TET targets were confined to a specific group of lineages and were rarely seen within the most common *emm* types in this study. This observation suggested that horizontal transfer of these resistant genes across lineages was uncommon (Varaldo et al., 2009). Although these genes were carried by various mobile genetic elements (data not shown), it is possible that these genetic elements confer detrimental fitness costs when transferred into normally non-resistant lineages. The proportion of erythromycin resistant isolates increased substantially between 2016 and 2017. The increase appeared to be caused by a general expansion of ERM-positive lineages relative to the ERM-negative lineages, with *emm* types 92 and 83 taking the leading role (Figure 2D). While changes in antibiotic consumption in certain populations have been associated with fluctuation in macrolide-resistance rates in *S. pyogenes*, (Murase and Yoshida, 1971; Bergman et al., 2004; Hsueh et al., 2005) it would be a unlikely to explain a 7% increase in ERM-positive isolates within 1 year. The increasing frequency of ERM-positive isolates that are also resistant to clindamycin, are a concern to public health. Clindamycin is regularly used to treat *S. pyogenes* infections in combination with β-lactams for patients with severe invasive disease (Allen and Moore, 2010; Stevens et al., 2014), and both clindamycin and erythromycin are alternative antibiotics for patients allergic to β-lactams (Bisno et al., 2002).

The WGS-based strain characterization enabled comprehensive detection of several previously characterized surface structure and virulence related features. A specific combination of these virulence features was usually unique to an *emm* type or a group of *emm* types and could be a contributing factor to the observed variation of virulence among different *emm* types. For example, isolates that were both PNGA3-positive and SOF-negative were solely represented by *emm* types 1, 12, and 3 while other SOF-negative *emm* types in Figure 3A (6, 84, 43, 191, and 101) were PNGA3-negative. These three *emm* types have been shown to be associated with higher risk of death due to iGAS infection (O’Loughlin et al., 2007; Thigpen et al., 2007; Nelson et al., 2016). WGS further enabled defining strain lineages within *emm* type based on genome-wide sequence variations. Three such lineages exhibited remarkable within-*emm* expansion between 2016 and 2017. These expansions could likely reflect development of local disease clusters or outbreaks that were initiated by ancestors of the individual lineages by chance. Alternatively, a within-*emm* expansion could suggest a selective advantage conferred by a novel bacterial feature other than *emm* type or strain features tightly associated with *emm* type. For example, all *emm92* isolates were erythromycin-resistant but only subgroup32 of *emm*92 isolates were expanding, suggesting neither the *emm* type nor erythromycin resistance was sufficient to explain the different dynamics. In fact, genomic sequence variations specific to each expanding lineage were identified in this study. The information could aid in both tracking future spread of a lineage and unveiling mechanisms underlying the emergence of a successful clade. In retrospect, monitoring of specific *emm* types in this manner could reveal key changes likely to be critical for strain success. For example, recombinational changes resulted in an altered the surface protein antigenic profile and increased toxin production by the *emm89* clonal complex. These changes led to the dramatic global emergence during the past 15 years (Beres et al., 2016). We have recently noted the appearance of 11 isolates of the recently reported M1_UK_ exotoxin over-producing clone, identifiable through a small set of characteristic base substitutions, that has dramatically emerged as an important cause of scarlet fever in the United Kingdom (Lynskey et al., 2019; Li et al., 2020). We will prospectively monitor this strain carefully within ABCs to determine if it becomes an important cause of invasive infections.

This study had several limitations. First, *emm* type distributions are known to vary substantially among different geographic locations (Steer et al., 2009). The broad category of “*sof*-positive *emm* types” when found in temperate or tropical countries often display completely unrelated lineages to their counterparts in North America or Western European countries (McGregor et al., 2004; Sakota et al., 2006; Bessen et al., 2018). Therefore, it remained to be demonstrated whether the theoretical vaccine coverage and the strain feature frequencies would be similar in regions outside the ABCs catchment areas. Second, the study period (24-month) was relatively short, which made it difficult to determine whether any lineage dynamic observed represented a temporary, stochastic fluctuation or a long-term, mechanistic change in strain virulence. Continued genomic surveillance is critical to understanding the impact of molecular events associated with emerging lineages causing iGAS disease in order to design more effective control measures. Third, in this study we mainly performed genomic characterization of invasive GAS isolates. Phenotypic testing of strain virulence and toxin productions was not performed, although genome data on antimicrobial susceptibility have been validated. In addition, the observations of this study were limited to invasive infections in the United States. Future studies are needed to compare these observations to characterization of non-invasive infections collected concurrently in time and place, and to gain better understanding of the dynamics of strain transmission within and between the different infection types. Non-invasive GAS infections also cause a large burden to human health. While the absolute reduction in non-invasive GAS infections from a vaccine will be great, impact of any decline in the considerable morbidity and mortality due to invasive infections will be non-trivial and important drivers of GAS vaccine policy. Additional experimental studies and phenotypic testing are needed to evaluate fitness changes associated with a strain. The study only examined a selected group of previously reported virulence factors. More in-depth sub-analyses of additional genetic factors with influence on virulence are needed in future studies.

In conclusion, findings of the study suggested that the overall population structure of invasive *S. pyogenes* in the United States was stable between 2016 and 2017, but expanding lineages, including antibiotic resistant ones, were evident. The stability of the population structure within ABCs over time has far-reaching implications. For example, the increasing resistance within ABCs was largely due to types that are included within the current experimental 30 valent vaccine. Surveillance of this nature will serve to distinguish non-significant alterations associated with emerging strains from the important ones. Further, the high-quality genomic sequences reported here provides an excellent foundation for other independent analyses in the field of GAS genomics.

## Data Availability Statement

The datasets generated for this study can be found in the NCBI BioProject PRJNA395240.

## Ethics Statement

The ABCs case reporting and isolate collection were reviewed in accordance with Centers for Disease Control and Prevention human research protection procedures and were determined to be non-research public health surveillance. The ABCs sites reviewed the protocol and obtained institutional review board approval where required.

## Author Contributions

YL designed the study, performed the analyses provided, and wrote the initial manuscript draft. SC performed, directed, and evaluated all whole genome sequencing data. BM developed the bioinformatics pipeline and provided periodic updates. JR, SM, and ZL performed whole genome sequencing. PS-V and RL provided phenotypic MIC data and analysis for all isolates from the Minnesota surveillance site. SV maintained *emm* typing test as well as performed conventional testing of selected isolates. SN and CV assisted in population-based epidemiologic data analysis and writing. BB and LM oversaw all laboratory operations. BB guided bioinformatics pipeline development. All authors reviewed the manuscript and provided constructive feedback.

## Conflict of Interest

The authors declare that the research was conducted in the absence of any commercial or financial relationships that could be construed as a potential conflict of interest.
