# Supplementary material for: Genomic Surveillance of Streptococcus pyogenes Strains Causing Invasive Disease, United States, 2016–2017
Source: Front Microbiol. 2020 Jul 24;11:1547. doi: 10.3389/fmicb.2020.01547 (PMC7396493; doi:10.3389/fmicb.2020.01547)
Supplement: Supplementary file 1 [file Presentation_1.pdf]

Supplementary Method

WGS-based AST Quality Control

Isolates recovered in the Minnesota ABCs site (n=591) were subjected to both WGS-based AST as part of the WGS analysis at the CDC's *Streptococcus* Laboratory and conventional phenotypic, culture-based AST at the Minnesota Public Health Laboratory for 14 antibiotics (Table S4). The results were compared to identify and quantify errors as a quality control measure. A WGS-based AST result was confirmed as correct and used as the final AST result if it agreed with the phenotypic AST result. When there was a discrepancy, a repeated phenotypic AST was performed for the isolate at the CDC's *Streptococcus* Laboratory and the mode value of the three AST results (initial phenotypic AST, WGS-based AST, and repeated phenotypic AST) was used as the final AST result. The result that differed from the final AST result was considered as an error. Criteria for acceptable quality included that the error rate of WGS-based AST results was 1) less than 5% and 2) not significantly higher than that of the initial phenotypic AST. For the 591 isolates and 14 antibiotics, initial phenotypic AST and WGS-based AST each generated 8274 results. A total of four discrepancy instances were observed in three isolates (Table S4). WGS-based AST results were acceptable with errors found in three instances (0.036%, 95% CI 0.0074% to 0.11%). One instance of an error was found for an initial phenotypic AST result (0.012%, 95% CI 0.0003% to 0.067%). Antibiotic-specific error proportions are shown in Table S4.

Table S1. Distribution of antibiotic resistance targets in invasive *S. pyogenes* isolates identified through the Active Bacterial Core surveillance (ABCs) system in the U.S. in 2016 and 2017

Antibiotic	Resistance target observed	Count (proportion, %)
**Clindamycin**	ERMT	253(6.98%)
	ERMB	205(5.65%)
	ERMTR	202(5.57%)
	ERM	5(0.14%)
	ERMB:LSAC	2(0.06%)
	23S3-C12T:ERMB	1(0.03%)
	23S3-C4T,C55T:ERM	1(0.03%)
	23S3-C4T,C55T:ERMT	1(0.03%)
	ERMB:ERMT:LSA	1(0.03%)
	ERMB:LSA	1(0.03%)
**Erythromycin**	ERMT	253(6.98%)
	ERMB	205(5.65%)
	ERMTR	202(5.57%)
	MEF	34(0.94%)
	ERM	5(0.14%)
	ERMB:LSAC	2(0.06%)
	23S3-C12T:ERMB	1(0.03%)
	23S3-C4T,C55T:ERM	1(0.03%)
	23S3-C4T,C55T:ERMT	1(0.03%)
	ERMB:ERMT:LSA	1(0.03%)
	ERMB:LSA	1(0.03%)
**Penicillin**	None	0(0)
**Ceftriaxone**	None	0(0)
**Vancomycin**	None	0(0)
**Chloramphenicol**	None	0(0)
**Daptomycin**	None	0(0)
**Linezolid**	None	0(0)

**Tetracycline**	TETM	699(19.28%)
	TETO	99(2.73%)
	TET	7(0.19%)
	TETL:TETM	2(0.06%)
**Levofloxacin**	PARC-S79F	42(1.16%)
	GYRA-S81F:PARC-S79Y	2(0.06%)
	GYRA-S81F:PARC-S79A	1(0.03%)
	GYRA-S81F:PARC-S79F	1(0.03%)
	GYRA-S81Y	1(0.03%)
	PARC-S79A	1(0.03%)
	PARC-S79Y	1(0.03%)
**Synercid**	None	0(0)
**Trimeth-sulfa**	None	0(0)
**Rifampin**	RPOB1-D476E	1(0.03%)

Table S2 Characteristic sequence differences between subgroup25 and non-subgroup25 *emm*82 invasive *Streptococcus pyogenes* isolates

**Gene**	**Product**	**Ref Seq**	**NT Position**	**Sequence in Subgroup25**	**Sequence in Non-Subgroup25**	**Synonymous Change**
slo	streptolysin O	YP_002997722.1	1399	C	T	Yes
prtF2	fibronectin binding protein	YP_279582.1	751	A	G	No
**csd1**	CRISPR-associated protein 2C	YP_002122935.1	772	C	T	No

Table S3 Characteristic sequence differences between subgroup171 and non-subgroup171 *emm*49 invasive *Streptococcus pyogenes* isolates

Gene	Product	Ref Seq	NT Position	Sequence in Subgroup25	Sequence in Non-Subgroup25	Synonymous Change
folP	dihydropteroate synthase	NP_269255.1	543	T	C	Yes
REF_00925	rhodanese-related sulfurtransferase	YP_005203847.1	802	T	C	No
bcaT	branched-chain amino acid aminotransferase	YP_598381.1	97	A	G	No

Table S4. Antibiotic-specific error rates of the WGS-based AST and phenotypic AST based on parallel testing of 591 isolates.

Antibiotic	WGS-based AST error (proportion)	Phenotypic AST error (proportion)
**ceftizoxime**	0 (0)	0 (0)
**cefoxitin**	0 (0)	0 (0)
**cefotaxime**	0 (0)	0 (0)
**cefazolin**	0 (0)	0 (0)
**ampicillin**	0 (0)	0 (0)
**penicillin**	0 (0)	0 (0)
**erythromycin**	1 (0.0017)[a]	0 (0)
**clindamycin**	1 (0.0017)[a]	1 (0.0017)[b]
**linezolid**	0 (0)	0 (0)
**tetracycline**	1 (0.0017)[c]	0 (0)
**ciprofloxacin**	0 (0)	0 (0)
**levofloxacin**	0 (0)	0 (0)
**daptomycin**	0 (0)	0 (0)
**vancomycin**	0 (0)	0 (0)

a. Isolate 20174040 was positive for the *ermT* gene target and was predicted as erythromycin resistant and clindamycin resistant by WGS-based AST. The initial phenotypic AST results were erythromycin susceptible and clindamycin susceptible. Final AST results were erythromycin susceptible and clindamycin susceptible. The error was possibly due to an inactive mutation in *ermT* gene or its promoter.

b. Isolate 20174040 was positive for the *ermB* gene target and was predicted as clindamycin resistant by WGS-based AST. The initial phenotypic AST result was clindamycin susceptible. Final AST result was clindamycin resistant. The error was possibly due to measurement uncertainty in phenotypic AST.

c. Isolate 20166450 was positive for the *tetM* gene target and was predicted as tetracycline resistant by WGS-based AST. The initial phenotypic AST result was tetracycline susceptible. Final AST result was tetracycline susceptible. The error was possibly due to an inactive mutation in *tetM* gene or its promoter.

Figure S1

A

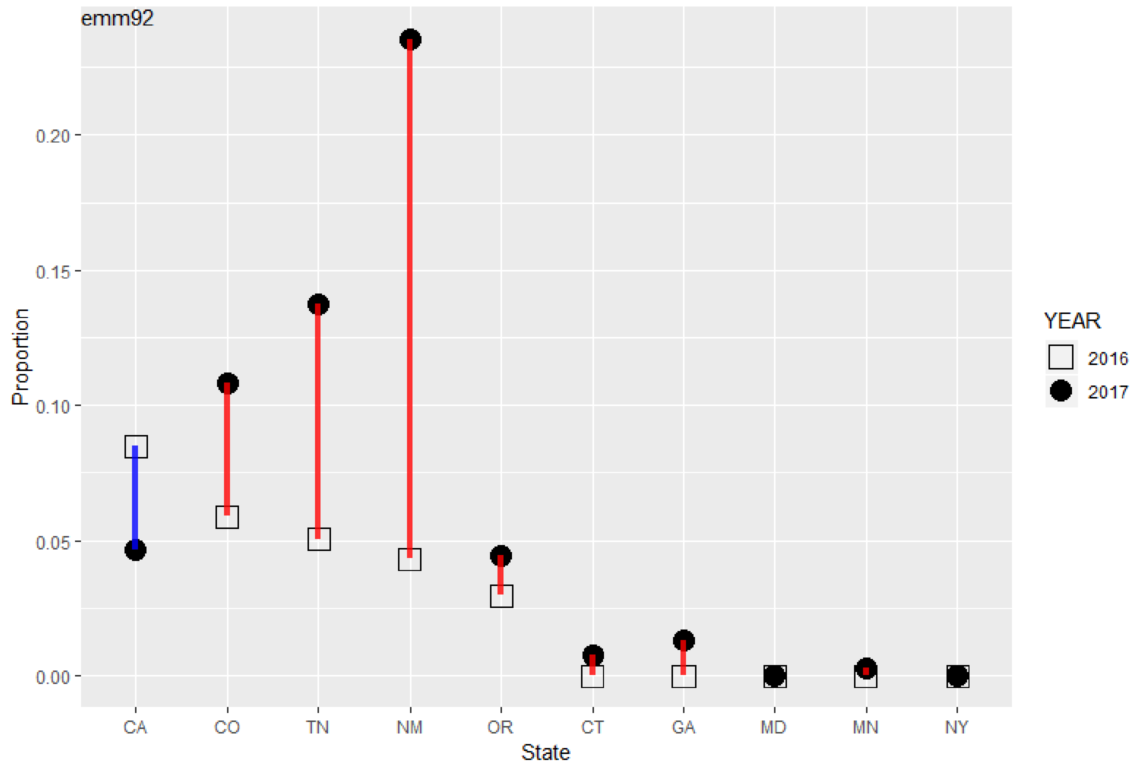
emm92
Proportion
0.00
0.05
0.10
0.15
0.20
CA
CO
TN
NM
OR
CT
GA
MD
MN
NY
State
YEAR
2016
2017

B

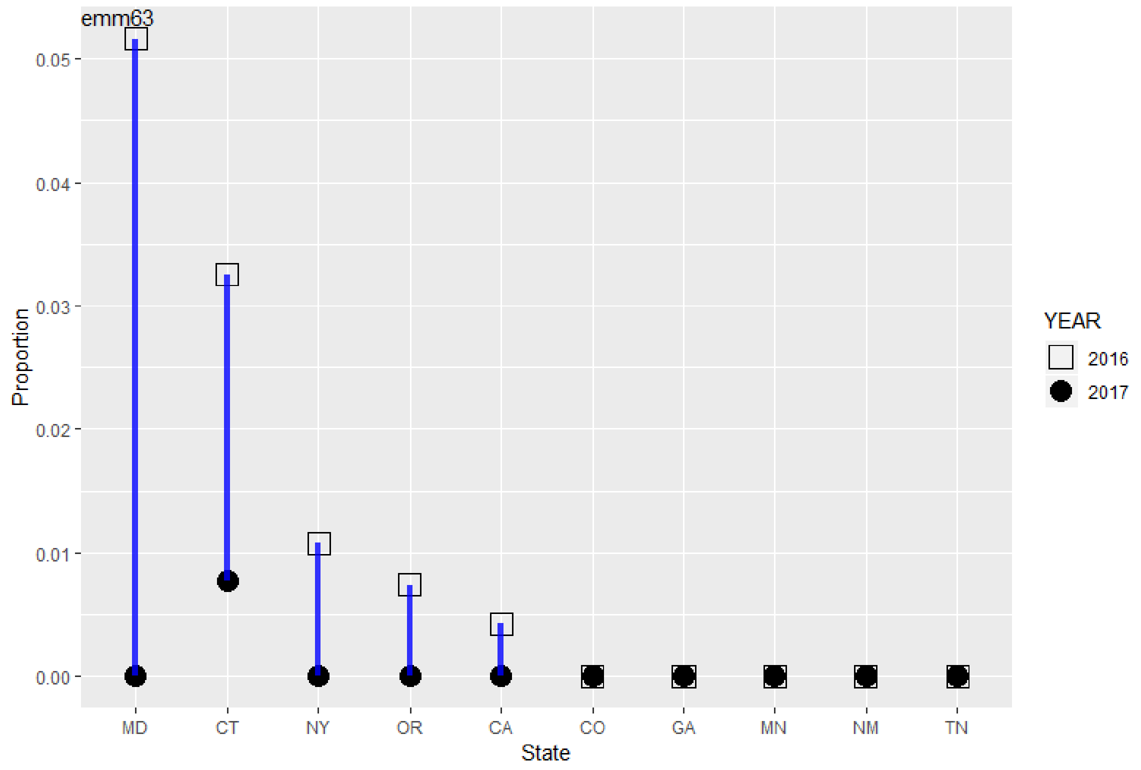
emm63
Proportion
0.00
0.01
0.02
0.03
0.04
0.05
MD
CT
NY
OR
CA
CO
GA
MN
NM
TN
State
YEAR
2016
2017

C

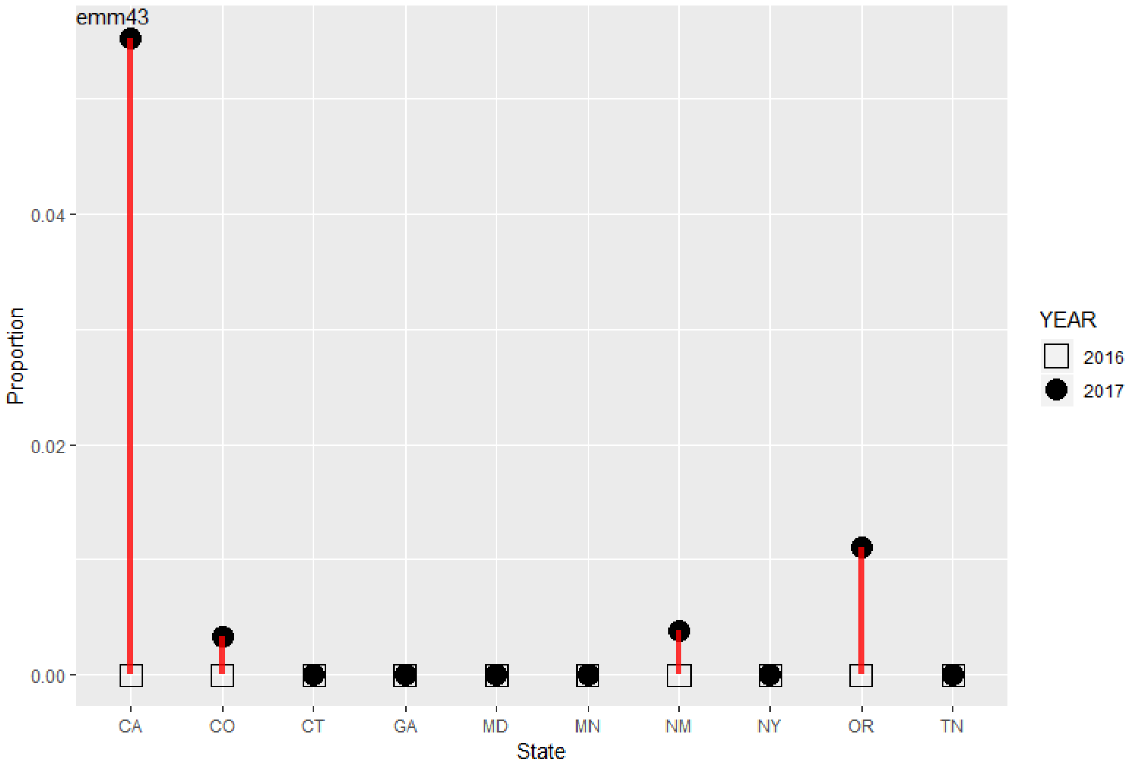

Figure S1. Change of *emm* relative frequency between 2016 (open square) and 2017 (solid circle) for *emm* types 92 (A), 63 (B), and 43 (C) by state. For each state, the proportion of isolates belong to the *emm* type in all isolates of that state in an indicated year is shown. The vertical bars indicate an increase (red) or decrease (blue) in proportion. Figure S1 is a descriptive illustration of data and no hypothesis was tested.
